# G2P datasets: a hub for genomic datasets for predictive modeling in plants and animals

**DOI:** 10.1093/g3journal/jkag001

**Published:** 2026-01-13

**Authors:** Fernando Aguate, Mark Watson, Harish Neelam, Yunxuan Deng, Jack Dekkers, Juan Pedro Steibel, Hao Cheng, Gustavo de los Campos

**Affiliations:** Department of Epidemiology and Biostatistics, Michigan State University, East Lansing, MI 48824, United States; Statistical and Quantitative Genetics, Department of Animal Science, University of California -Davis, Davis, CA 95616, United States; Department of Epidemiology and Biostatistics, Michigan State University, East Lansing, MI 48824, United States; Statistical and Quantitative Genetics, Department of Animal Science, University of California -Davis, Davis, CA 95616, United States; Department of Animal Science, Iowa State University, Ames, IA 50011, United States; Department of Animal Science, Iowa State University, Ames, IA 50011, United States; Statistical and Quantitative Genetics, Department of Animal Science, University of California -Davis, Davis, CA 95616, United States; Department of Epidemiology and Biostatistics, Michigan State University, East Lansing, MI 48824, United States; Department of Statistics and Probability, Michigan State University, East Lansing, MI 48824, United States; Institute for Quantitative Health Science and Engineering, Michigan State University, East Lansing, MI 48824, United States

**Keywords:** genomic prediction, plant breeding, animal breeding, SNP data, open-access repository, genomic research, data sharing, FAIR

## Abstract

We present G2P Datasets, a novel, open-access repository of publicly available genomic datasets for plants and animals. G2P Datasets currently hosts more than 100 public genomic datasets, meticulously compiled from diverse publications. The repository hosts meta-data (including digital object identifier for the hosted datasets) and scripts that can be used to download and read each dataset hosted into an R-environment. Scientists can submit new datasets by completing an online form. Additionally, many of the curated datasets are accessible through Kaggle and through links that allow rapid download of formatted R-objects. A searchable database of meta-data allows users to search through datasets for more than 60 species of plants and animals in a variety of traits and sample sizes. The repository is accessible via a web app interface with a catalog and clear instructions to contribute by adding new datasets and through GitHub. By unifying genomic and phenotypic meta-data into a navigable platform, we aim to facilitate genome-to-phenotype research in plant and animal genetics. This article provides an overview of the repository's content, organization, and utility.

## Teaser

Aguate *et al*. present G2P Datasets, a novel, open-access repository of publicly available genomic datasets for plants and animals. G2P Datasets currently hosts more than 100 public genomic datasets from diverse publications. The repository hosts meta-data and scripts that can be used to download and read datasets hosted into an R-environment. A searchable database of meta-data allows users to search through datasets for more than 60 species of plants and animals in a variety of traits and sample sizes. By unifying genomic and phenotypic meta-data into a navigable platform, the authors aim to facilitate genome-to-phenotype research in plant and animal genetics.

## Introduction

The rapid advancement of genomic technologies has led to an exponential growth in the generation of genomic and phenotypic data, enabling the development of sophisticated predictive models for plant and animal breeding. Analysis of datasets from traits, environments, populations, and species holds immense potential for enhancing our understanding of complex traits and accelerating genetic gains for species and populations of agronomic importance. Every year, thousands of studies involving quantitative genetics analyses of plant and animal data are published. Increasingly, scientific journals encourage—and some mandate—making the data used in those studies publicly available. Moreover, many funding agencies and journals require research data to be made public following the FAIR (findable, accessible, interoperable, and reusable) principles (Wilkinson et al. 2016).

Although a vast number of datasets containing genomic data have been published, several issues often hinder their reuse. The datasets are often presented in a diverse range of formats and are stored in different locations, ranging from software packages and public repositories to supplementary files in publications. To facilitate data accessibility, reproducibility, and collaborative research, we developed an extensive, open-access repository of meta-data to access genomic datasets, with scripts to read the data, unify format, and run predictive models that are commonly used in plant and animal breeding. The vision for this project is to enhance access to existing genomic resources, with a primary—though not exclusive—focus on plant and animal breeding. While comprehensive datasets such as UK Biobank (ukbiobank.ac.uk), The 1000 Genomes Project (1000genomes.org), and the European Genome-Phenome Archive (ega-archive.org) are available for human genomics, comparable resources for plants and animals remain fragmented and incomplete.

Currently, G2P Datasets references more than 100 original datasets comprising single-nucleotide polymorphism (SNP) genotypes, phenotypic data, and genetic maps for more than 60 animal and plant species. Meta-data from diverse publications were meticulously collected, encompassing a wide range of traits and species, to provide researchers with a broad and representative resource for genomic predictive modeling. While other projects have compiled genomic datasets (Morales et al. 2022; Chen et al. 2024), to our knowledge, this is the first attempt to consolidate such an extensive collection of genomic datasets across plant and animal breeding applications and to provide scripts to read and format datasets that can be readily used in genomics research.

G2P Datasets is organized in a user-friendly manner, allowing researchers to easily search, access, download, and upload genomic datasets. Unlike existing databases for genomic data, our repository does not store the original datasets themselves. Instead, datasets are represented by R-code to access data in situ via existing databases. This framework avoids redundancy in data storage, guarantees data integrity because data sources are not modified, and allows G2P Datasets to scale with minimal memory resources. For each dataset listed in the repository, we have meta-data that enables performing searches and accessing to relevant information, such as sample size, a short description of the research purpose, the digital object identifier (DOI), a citation to the article in which it was published, and a title for the dataset. This information not only helps researchers to quickly identify datasets of interest but also ensures proper attribution and citation of the original sources. Linked to each dataset, we provide R-scripts that can be used to read and format the downloaded data into R-objects that can be readily used in analyses.

G2P Datasets is accessible through a GitHub repository (https://github.com/QuantGen/G2P-Datasets) and through a Shiny web app (https://mtwatson.shinyapps.io/G2P-datasets/) featuring a searchable database. This interactive tool enables researchers to search for datasets and learn about their features before downloading them, thereby saving time and effort. The app also provides access to links from where each dataset can be downloaded and R-scripts that can be run to format the data into R-objects that are readily usable. To facilitate user contributions of new datasets, we include an electronic form in the G2P Datasets Shiny app that allows members of the community to submit new datasets. The app can also be downloaded from GitHub (https://github.com/mtwatso2-eng/G2P_dataset_app) to be executed locally, offering an alternative way to access this resource.

To illustrate some of the utilities that G2P Datasets can offer, we present a benchmark of commonly used genomic prediction models using a subset of hosted datasets (selected based on the data included and sample size). For these datasets, in addition to the meta-data included for all the datasets, we also offer analysis scripts and links to download the curated datasets directly from the repository and through Kaggle (https://www.kaggle.com/organizations/ag2p-disc/datasets).

## Materials and methods

To populate the database, we conducted a web-search to identify publicly available genomic datasets suitable for genomic prediction modeling in plants and animals. Our search focused on plant and animal data and excluded fungi, bacteria, protist, and human data. We searched online repositories, Supplementary materials of published articles, and data archives, and we filtered out data archives that require submitting a data request or a subscription, in an effort of maximizing data accessibility. The search was performed using a combination of keywords related to genomic prediction, GWAS, plant breeding, animal breeding, SNP data, and phenotypic data.

We included in the repository datasets that (i) were from plants and animals of agricultural or veterinary importance, (ii) included DNA genotypes linked to phenotypes, and (iii) were publicly available and downloadable without requiring registration or consent.

We do not claim to have included all the datasets that meet these criteria in the database at this point in time. Rather, our objective was to populate G2P Datasets with a reasonably large number of datasets. Members of the scientific community can then use the available electronic form to help populate the repository with additional datasets that meet these criteria.

### Data types

The datasets listed in the repository may contain a variety of data types to accommodate diverse research needs in genomic predictive modeling. The primary data types include:

- DNA genotypes stored in formats such as *.vcf*, *.bed*, *.rds*, *.txt*, *.csv*, or *.ped* extensions.
- Phenotypic data (either quantitative or qualitative), including single or multiple individual phenotypes, as well means (possibly adjusted by experimental factors). In some cases, the phenotype file also included information about the experiments from which the data were collected. Phenotype data are typically stored in tables in ASCII (.*txt* or .*cvs* extensions), *.xls*, or using software-specific binary formats (e.g. *.rds*).
- Many datasets also include genetic maps for the SNPs included in the genotype files, while some datasets offer pedigree and/or genomic relationships instead of or along with the individual genotype data.
- Likewise, some datasets include meta-data describing management or environmental conditions under which the phenotypic data were collected.

Some datasets have a partial overlap of genotypes and come from the same species; we could have merged those datasets but opted not to do so because the spirit of G2P Dataset is to help researchers find datasets and streamline access without modifying the original data.

### Data formatting

To streamline analyses, for each dataset, we developed R-scripts that, starting from the file format provided by the source, produce R-objects (data-frames for phenotypes, matrices for genotype data) that are readily useable within an R-environment. These scripts can be obtained from the Web application. To maintain the integrity of the original data and allow researchers flexibility to perform their own quality control and filtering steps, the scripts that we provide preform minimal data pre-processing.

### Web application

We developed a Web Application interface for the G2P Datasets repository, built using the R package *Shiny* (https://shiny.posit.co/) and is available at https://mtwatson.shinyapps.io/G2P-datasets/. This application facilitates user interactions with the project through two main modules: “Browse Datasets” and “Add Dataset.” Their functions are described below:

- *Browse Datasets*: This module displays a searchable table of datasets that can be filtered based on meta-data features, which include a short title, the number of individuals that were genotyped, the number of DNA markers genotyped, whether there is phenotypic data available, and links to articles and datasets. The search tool will also filter by information contained in the abstract, title, scientific name, and/or common name; for example, searching for the word “environments” filters several datasets that potentially contain multiple environments. When a desired dataset is selected, relevant information about the dataset (the title of publication, DOI, and the scientific and common name of the species) is displayed, along with collapsible menus that can be selected to gain access to the abstract of the publication, the R-scripts that we developed for the selected dataset, citation information, and links to download the data.
- *Add dataset*: This module includes a web-form that users can complete to submit new datasets, with clear instructions in the “About the project” section of the Web Application. The form collects information about the publication (including a DOI to the data), sample size, and the number of DNA markers, along with an entry where scripts to read the data can be uploaded. To enhance dataset meta-data, a OpenAlex API is used to retrieve supplementary information, such as the article's abstract and web link. Before submission, users can preview the meta-data to ensure accuracy and are encouraged to complete any missing information to enhance the meta-data's comprehensiveness. Once finalized, the contributor downloads a folder that needs to be uploaded to the GitHub repository.

### Benchmarks

To showcase an application using large and diverse genomic data, we present a genomic prediction benchmark involving 42 datasets that are currently available in G2P Datasets. We benchmarked the cross-validation prediction performance of four genomic prediction models, including three linear models, GBLUP (Van Raden 2008), BayesB (Meuwissen et al. 2001), tree boosting model, XGBT (Chen and Guestrin 2016), and a Reproducing Kernel Hilbert Spaces regression using three Gaussian kernels, GKAD (de los Campos et al. 2010). To benchmark each dataset, SNPs with minor allele frequency smaller than 0.03 were removed. The remaining uncalled genotypes were imputed with the mean genotype of the corresponding SNP in each dataset.

Following Couto-Alves et al. (2019), for the GKAD, we first computed a distance matrix based on a (linear) genomic relationship matrix and then derived from it three Gaussian kernels using bandwidth parameters of 0.5, 1, and 1.5. Thus, the GKAD model included three random effects, each with a different kernel matrix.

To evaluate prediction performance, for each dataset and trait, we conducted 100 training–testing partitions, each time randomly assigning 80% of the data to training and 20% to testing. Thus, for each dataset, trait, and model, we had 100 estimates of the correlation between predictions and observed phenotypes in the testing data. From these results, we report the average prediction correlation by model, trait, and dataset. In addition to these training–testing evaluations, we also fitted the GBLUP model to each of the traits in each of the (full) datasets to estimate genomic heritability (de los Campos et al. 2015) for each trait and each dataset.

All analyses were done in the R software (R Core Team 2024); the linear models and the RKHS regressions were fitted using the R package *BGLR* (Perez and de los Campos 2014), and XGBT regressions were fitted using the *xgboost* R package (Chen and Guestrin 2016). The genomic relationship matrices were derived using the *getG()* function of the *BGData* R package (Grueneberg and de los Campos 2019). Plots were done using *ggplot2* (Wickham 2016).

The 42 formatted/edited datasets used in this benchmark were saved as binary R-files and made available in *.rdata* format through Kaggle and in the GitHub repository of G2P Datasets. Therefore, studies proposing new models can easily use these datasets to benchmark the proposed methodologies against standard ones.

## Results and discussion

**Fig. 1.**
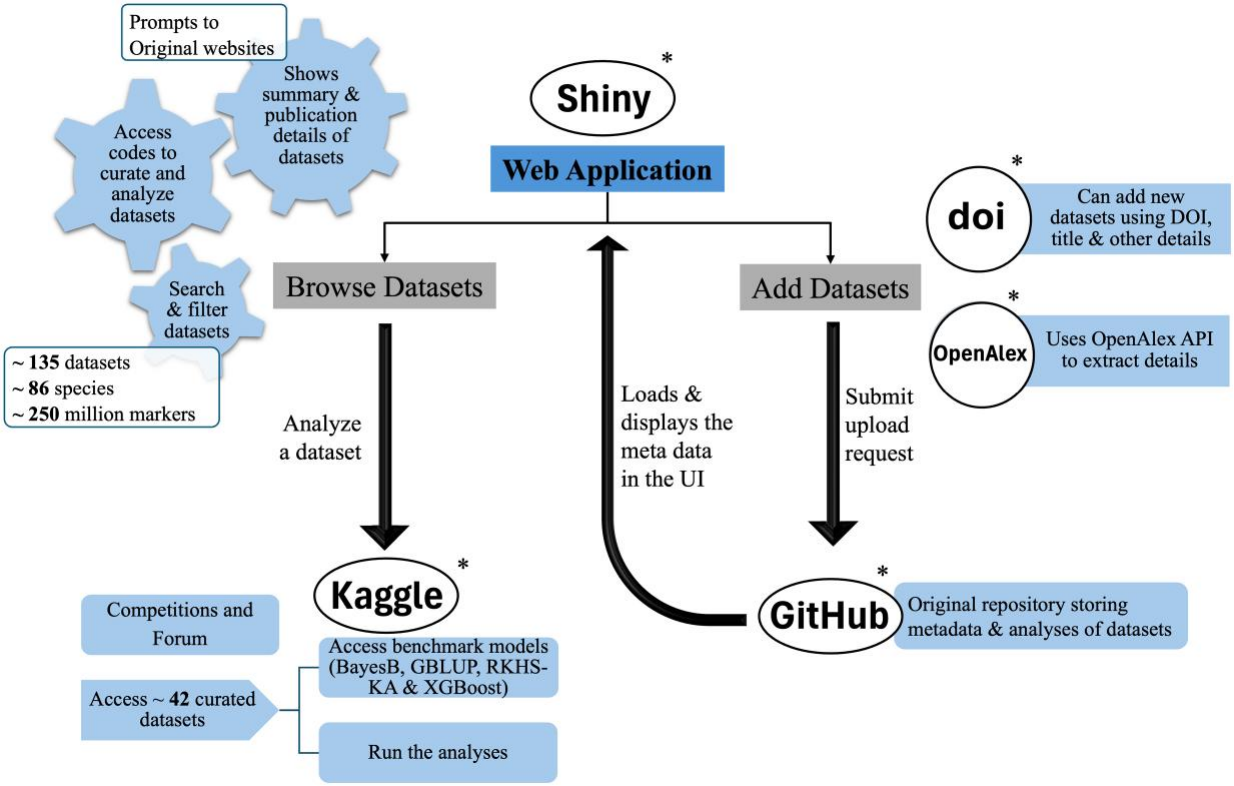
Graphical representation of the G2P datasets project. The core of the project (where meta-data is stored) is a GitHub repository. A Web Application provides a searchable database (“Browse Datasets”) and an electronic form to contribute new datasets (“Add Datasets”). A subset of the datasets included in the database are also available (as R binary objects) in Kaggle. *Outside entities.

Figure 1 illustrates the G2P Datasets project, including a GitHub repository (hosting meta-data for each of the datasets), a Web Application, and a subset of the datasets (in binary R-format) in Kaggle.

**Fig. 2.**
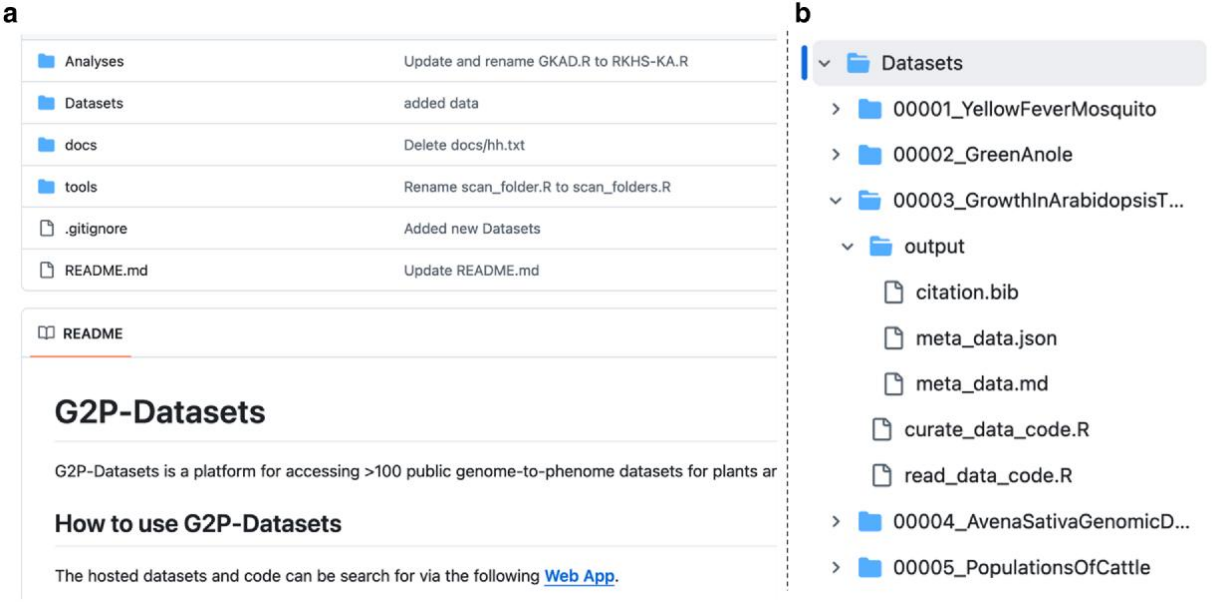
The GPDatasets GitHub repository includes meta-data for each of the datasets hosted in the project (*Dataset* folder), templates of the scripts used for the benchmarks (analyses), tools, and documentation (panel a). Each dataset has a folder with scripts to read and perform quality control on the data and meta-data (including a citation file and meta-data in *json* and md format, panel b).

As indicated in Fig. 2, the GitHub repository includes the folder Datasets, which contain subfolders corresponding to each dataset; each of these subfolders hosts the meta-data, the R-scripts, and the curated version of the data, if available.

The Web Application reads the meta-data from the repository in GitHub, provides a searchable database through the “Browse Dataset” module, and facilitates contribution of new datasets to the project through the “Add Datasets” module. The Web Application is the main User Interface (UI) to browse through all the datasets and R-scripts.

**Fig. 3.**
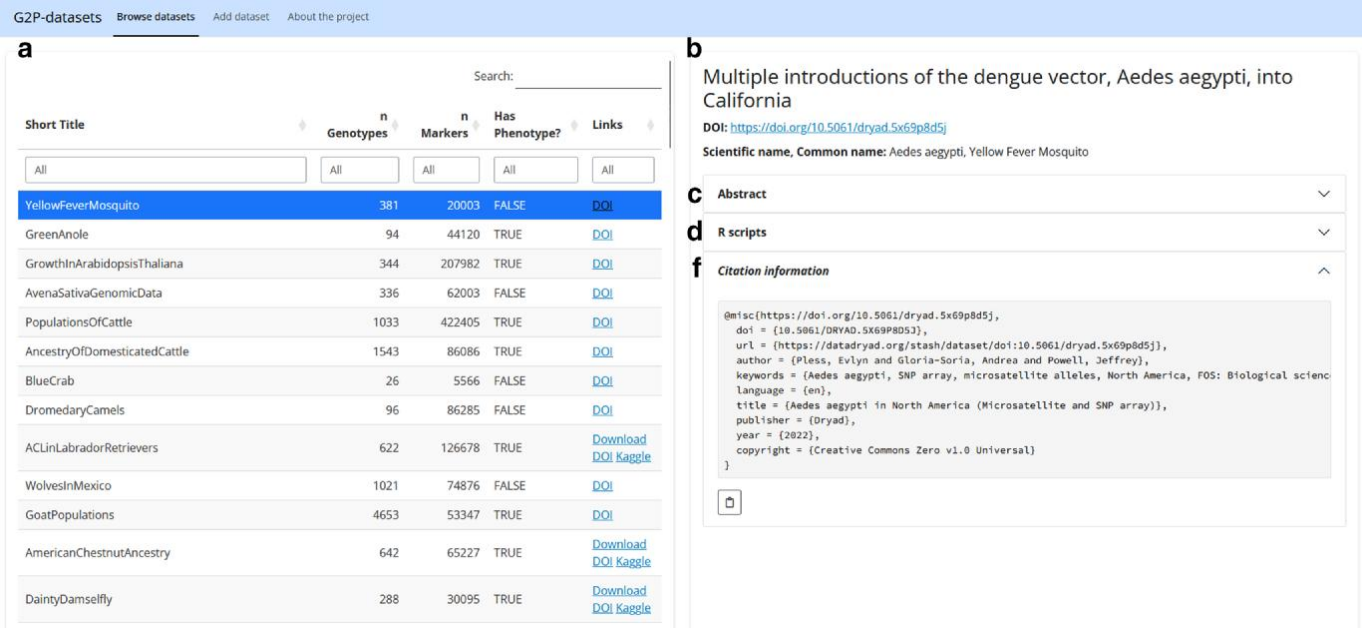
Overview of the web app. Panel a) displays the searchable table of datasets; Panel b) displays details of the selected dataset, including Title (b), DOI, Abstract (c), Scripts (d), and citation information (f).

The UI layout of the Web Application is shown in Fig. 3. The Web Application is split into two main panels. Panel A in Fig. 3 displays the searchable meta-data table, allowing users to filter datasets by short name, number of genotyped individuals, number of DNA markers, presence of phenotypic data, and tags. A global search box found at the top-right corner of this block is also available for users to filter datasets by keywords. Once the user selects one dataset of the table in panel A, panel B in Fig. 3 shows the publication information, meta-data, and scripts pertinent to the selected dataset. Within panel B, there is a header for a simplified title, a corresponding DOI, and scientific and common names for the species of the study. Collapsible sub-blocks (c), (d), and (e) contain, respectively, the article's abstract; R-scripts to read, curate, and run a statistical model on the data; and the citation information. When available, a curated dataset can be directly downloaded from a download button in the header of panel B.

The header of the Web Application (panel A in Fig. 3) includes a tab to display the *Add Dataset* module, which can be used to submit a new dataset to the repository. In this module, a project contributor must provide and validate meta-data for a dataset that is not currently in the repository. After downloading the formatted meta-data folder, the contributor must add the folder to the GitHub repository of the G2P Datasets project. A repository maintainer will review the dataset to ensure it is reachable with the links provided and make the dataset available in the Web Application. More instructions about how to upload new datasets can be found in the *About the project* section.

Instructions to download a raw dataset can be found within the R-Scripts sub-panel (d), which also contains copy-to-clipboard buttons to facilitate copying the scripts into any Integrated Development Environment (IDE) for R programming. Scripts to run the different predictive models are also proposed within this sub-panel.

### Benchmarks

**Fig. 4.**
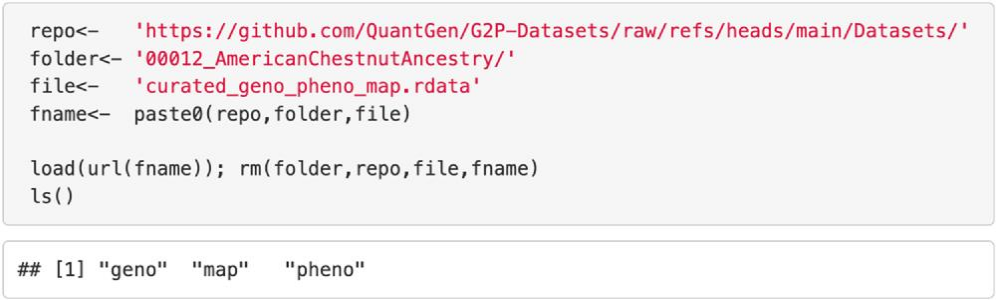
An example R-script to load a dataset from the GitHub repository into an R-environment. To load a different dataset only the folder name needs to be changed. Only the datasets used in the benchmark are provided as R binary objects in the repository.

As stated earlier, we used 42 datasets for the benchmarks; these datasets can be accessed (as binary R-objects) either from Kaggle (https://www.kaggle.com/organizations/ag2p-disc, a free-access platform where learners, researchers, and developers can connect with the community to share scripts, data, and feedback) or by downloading directly from the GitHub repository using a script as the one displayed in Fig. 4.

**Fig. 5.**
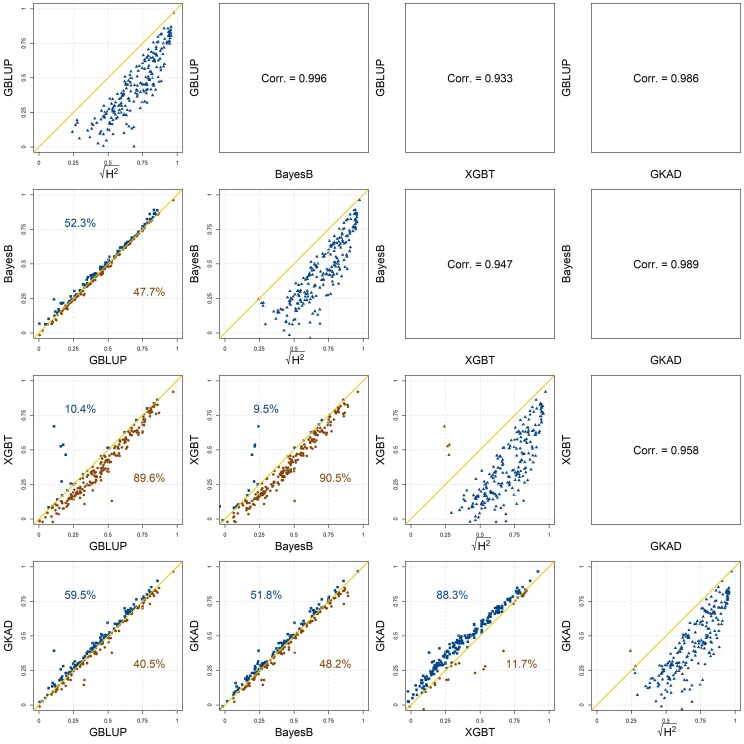
Plots of the average prediction correlation (over 100 training–testing partitions) by trait, dataset, and method against the square root of the heritability on the diagonal for different prediction models. The plots below the diagonal display the prediction accuracy achieved by one model vs the accuracy achieved by another model. The percentage values above the 45-degree line indicate the proportion of cases the model in the *y*-axis outperformed the model displayed in the *x*-axis. Mirroring this percentage, the value below the 45-degree line corresponds to the cases when the model in the *x*-axis outperformed the one in the *y*-axis. The panels above the diagonal show Pearson's correlation between prediction accuracies for the models in the *x*- and *y*-axes, calculated across trait–dataset cases.

Figure 5 shows the results of the benchmarks. The plots in the diagonal panels display the average prediction correlation achieved by the model in the *y*-axis vs the square root of the heritability (*x*-axis). The heritabilities ranged from low (∼0.06, corresponding to a square-root heritability of ∼0.25) to moderately high (∼0.8, corresponding to a square-root heritability near 0.9). As expected, the prediction correlations are lower than the square root of heritabilities but they increased with heritability. For a more in-depth discussion of the factors affecting prediction accuracy, we refer to Daetwyler et al. (2013) and Kaler et al. (2022). The data used to generate the figure are available as Supplementary material (see Supplementary Table 1).

Most of the points in the below-diagonal plots are near the 45-degree line, indicating that the prediction accuracies achieved by the different models were relatively similar. However, on average, the kernel model (GKAD) performed better than the other models. This model outperformed GBLUP in 60% of the trait–dataset combinations; however, the difference in the magnitude of the prediction correlations was small. At the other end, XGBT was the worst performing model and linear models outperformed XBGT in ∼90% of the trait–dataset combinations.

## Final remarks

Data sharing and collaboration are essential to advance open science; this is especially important in genomic prediction modeling. In the last two decades, a large number of genomic datasets have been developed, and many of these were shared in the public domain, usually linked to scientific publications. The G2P Datasets initiative aims to facilitate access to the large collection of datasets for investigations and benchmarking of genomic prediction models. The repository is populated with more than 100 datasets and provides a platform for scientists to contribute with more datasets.

## Supplementary Material

jkag001_Supplementary_Data

## Data Availability

All the data and the analyses scripts used to produce benchmarks can be downloaded from https://github.com/QuantGen/G2P-Datasets. The web application code and repository used in this study have been permanently archived in Zenodo and are publicly available (repository, https://doi.org/10.5281/zenodo.17604233; web application, https://doi.org/10.5281/zenodo.17604237). Supplemental material available at G3 online.

## Web resources

Datasets catalog: https://mtwatson.shinyapps.io/G2P-Datasets/

Github repository: https://github.com/QuantGen/G2P-Datasets

Kaggle organization: https://www.kaggle.com/organizations/ag2p-disc

The Agricultural Genome to Phenome—Data Integration, Sharing, and Collaboration (AG2P-DISC) website provides access to these resources: https://ag2p-disc.github.io/
